# The Use of Oral Amino‐Bisphosphonates and Coronavirus Disease 2019 (COVID‐19) Outcomes

**DOI:** 10.1002/jbmr.4419

**Published:** 2021-08-22

**Authors:** Luca Degli Esposti, Valentina Perrone, Diego Sangiorgi, Margherita Andretta, Fausto Bartolini, Arturo Cavaliere, Andrea Ciaccia, Stefania Dell'orco, Stefano Grego, Sara Salzano, Loredana Ubertazzo, Adriano Vercellone, Davide Gatti, Angelo Fassio, Ombretta Viapiana, Maurizio Rossini, Giovanni Adami

**Affiliations:** ^1^ CliCon S.r.l. Health Economics & Outcomes Research Bologna Italy; ^2^ UOC Assistenza Farmaceutica Territoriale, Azienda ULSS 8 Berica Vicenza Italy; ^3^ Dipartimento Farmaceutico, USL Umbria 2 Perugia Italy; ^4^ UOC Farmacia Aziendale, ASL Viterbo Italy; ^5^ Dipartimento Farmaceutico, ASL Foggia Italy; ^6^ UOC Farmaceutica Territoriale, ASL Roma 6 Rome Italy; ^7^ Dipartimento Tecnico‐Amministrativo, ASL 3 Genovese Genova Italy; ^8^ UOC Farmacia Territoriale, ASL Roma 4 Rome Italy; ^9^ Dipartimento Farmaceutico, ASL Naples 3 Sud Naples Italy; ^10^ Rheumatology Unit University of Verona Verona Italy

**Keywords:** BISPHOSPHONATES, COVID‐19, INFECTION RISK, OSTEOPOROSIS, SARS‐COV‐2

## Abstract

The determinants of the susceptibility to severe acute respiratory syndrome‐coronavirus‐2 (SARS‐CoV‐2) infection and severe coronavirus disease 2019 (COVID‐19) manifestations are yet not fully understood. Amino‐bisphosphonates (N‐BPs) have anti‐inflammatory properties and have been shown to reduce the incidence of lower respiratory infections, cardiovascular events, and cancer. We conducted a population‐based retrospective observational cohort study with the primary objective of determining if oral N‐BPs treatment can play a role in the susceptibility to development of severe COVID‐19. Administrative International Classification of Diseases, Ninth Revision, Clinical ModificationI (ICD‐9‐CM) and anatomical‐therapeutic chemical (ATC) code data, representative of Italian population (9% sample of the overall population), were analyzed. Oral N‐BPs (mainly alendronate and risedronate) were included in the analysis, zoledronic acid was excluded because of the low number of patients at risk. Incidence of COVID‐19 hospitalization was 12.32 (95% confidence interval [CI], 9.61–15.04) and 11.55 (95% CI, 8.91–14.20), of intensive care unit (ICU) utilization because of COVID‐19 was 1.25 (95% CI, 0.38–2.11) and 1.42 (95% CI, 0.49–2.36), and of all‐cause death was 4.06 (95% CI, 2.50–5.61) and 3.96 (95% CI, 2.41–5.51) for oral N‐BPs users and nonusers, respectively. Sensitivity analyses that excluded patients with prevalent vertebral or hip fragility fractures and without concomitant glucocorticoid treatment yielded similar results. In conclusion, we found that the incidence of COVID‐19 hospitalization, intensive care unit (ICU) utilization, and COVID‐19 potentially related mortality were similar in N‐BPs–treated and nontreated subjects. Similar results were found in N‐BPs versus other anti‐osteoporotic drugs. We provide real‐life data on the safety of oral N‐BPs in terms of severe COVID‐19 risk on a population‐based cohort. Our results do not support the hypothesis that oral N‐BPs can prevent COVID‐19 infection and/or severe COVID‐19; however, they do not seem to increase the risk. © 2021 The Authors. *Journal of Bone and Mineral Research* published by Wiley Periodicals LLC on behalf of American Society for Bone and Mineral Research (ASBMR).

## Introduction

Coronavirus disease 2019 (COVID‐19) can be life threatening due to acute respiratory distress (ARDS), especially in the elderly and in patients with associated comorbidities.^(^ ^1^ ^)^ In Italy, as of January 28, 2021, more than 2.5 million of cases of severe acute respiratory syndrome‐coronavirus‐2 (SARS‐CoV‐2) infections had been recorded with a death toll counting 87,000 casualties related to COVID‐19.^(^ ^2^ ^)^ There is increasing evidence that the host immune system overreaction to SARS‐CoV‐2 plays a fundamental role on the development of severe manifestations of the disease,^(^ ^3^ ^)^ specifically in what is known as the cytokine storm related to COVID‐19.^(^ ^3^ ^)^ However, albeit multiple immunological patterns have been investigated to explain the susceptibility to severe COVID‐19, none of the proposed pathways alone is likely to fully explain the predisposition to the abnormal immune response to SARS‐CoV‐2 infection.^(^ ^4^ ^)^ Indeed, the disease course of COVID‐19 is unpredictable as testified by the fact that most patients with SARS‐CoV‐2 infection have an asymptomatic or paucisymptomatic disease course,^(^ ^1^ ^)^ not all the subjects infected develop severe infections, and only a small proportion of them require intensive care.^(^ ^1^ ^)^ Therefore, there are other, still unidentified, external factors that regulate the response to SARS‐CoV‐2 infection. Unrevealing the factors that lead to an uncontrolled inflammatory response to the SARS‐CoV‐2 might help target the interventions in the early stages of the disease, especially in high‐risk populations.

Bisphosphonates largely dominates the treatment of primary and secondary osteoporosis, in both female and male individuals. Bisphosphonates, and in particular amino bisphosphonates (N‐BPs), are indeed widely used in the population, with more than 30 million doses prescribed every year and approximately 5 million patients treated chronically in the United States. N‐BPs have proven effects on immune cells. In particular, clinical studies on human beings have shown that N‐BPs can reduce the amount of circulating γδ T cells, with hypothesized immune‐regulating effects.^(^ ^5^ ^)^ Indeed, N‐BPs have been listed among the potential factors related to an attenuated infection; in particular, a small observational study has recently shown that the incidence of SARS‐CoV‐2 infection was somehow lower in patients treated with zoledronic acid as compared to the general population.^(^ ^6^ ^)^

The aim of the present study was to determine if oral N‐BPs treatment is associated with the incidence of COVID‐19 hospitalization, of COVID‐19 hospitalization in the intensive care unit (ICU), and of mortality potentially related to COVID‐19 at a population level.

## Patients and Methods

### Data source

Data analyzed were extracted from the administrative databases of a sample of Local Health Units (LHUs) geographically distributed throughout the Italian territory and covering approximately the 9% of Italian population. These healthcare administrative databases are large repositories of data on healthcare systems that are routinely collected and provide a variety of already stored data with an ongoing collection process. Administrative data include healthcare services provided by Italian National health Service (INHS); therefore, they hold information meant to be used for administrative purposes in order to track the economic flows from the INHS to the healthcare providers for reimbursement purposes. Specifically, to perform the analysis, the following databases were used: demographic database to collect demographic characteristics (age, sex, and date of death); pharmaceuticals database for data regarding drugs dispensed as date of prescription, number of packages and units per package, anatomical‐therapeutic chemical (ATC) code; and hospitalization database for discharge diagnosis and procedure codes based on the International Classification of Diseases, Ninth Revision, Clinical Modification, ICD‐9‐CM; outpatient specialist services database that includes data on diagnostic tests and specialist visits. Each database contains the anonymous univocal patient ID (numeric code) that allows the data‐linkage across all different databases to create an individual clinical and pharmacological profile for each patient.

### Study design

A retrospective observational cohort study was conducted. All health‐assisted individuals of the LHUs involved were enrolled (around 5.4 million people) between January 2019 and June 2020. Therefore, data covered the first pandemic wave of COVID‐19. The following treatments were tracked during 2019: bisphosphonates (bisphosphonates listed under the ATC code M05BA and bisphosphonates combination listed under the ATC code M05BB); respiratory drugs (ATC code R03, drugs for obstructive airway diseases; ATC code R03A, adrenergics and inhalants; ATC code R03B, other drugs for obstructive airway diseases and inhalants; ATC code R03C, adrenergics for systemic use; and ATC code R03D, other systemic drugs for obstructive airway diseases); antihypertensives (C02, C03, C07, C08, C09); statins (C10A); antidiabetics (ATC code A10A, insulins and analogues; ATC code A10B, blood glucose lowering drugs, excluding insulins; and ATC code A10X, other drugs used in diabetes); antidepressants (N06A); corticosteroids (H02); other anti‐osteoporotic therapies (ATC code M05BX, other drugs affecting bone structure and mineralization; ATC code H05AA, parathyroid hormones and analogues; ATC code H05BA, calcitonin preparations; and ATC code G03XC, selective estrogen receptor modulators). Hospitalizations were analyzed looking back at all period before January 2020 (data availability starting from 2010) and regarded respiratory system (ICD‐9‐CM code 460–466, acute respiratory infections; ICD‐9‐CM code 470–478, other diseases of the upper respiratory tract; ICD‐9‐CM code 480–488, pneumonia and influenza; ICD‐9‐CM code 490–496, chronic obstructive pulmonary disease and allied conditions; ICD‐9‐CM code 500–508, pneumoconioses and other lung diseases due to external agents; and ICD‐9‐CM code 510–519, other diseases of respiratory system), neoplasms (ICD‐9‐CM code 140–199); and cardiovascular and cerebrovascular conditions (ICD‐9‐CM code 410–459). Presence of previous vertebral or hip fracture (ICD‐9‐CM 805, 806, 820, 821) was investigated as well.

Patients treated with bisphosphonates were randomly matched (1:1 ratio) for age, sex, and for each variable described in the previous paragraph (presence of treatments other than bisphosphonates and hospitalizations) with all the health‐assisted population without this treatment.

Starting from the COVID‐19 outbreak, the event of COVID‐19 hospitalization was identified within the hospitalization database by presence of ICD‐9‐CM code 078.89, COVID‐19 hospitalization in intensive care unit (ICU) by presence of ICD‐9‐CM code 078.89, and ICU department code: 49. All‐cause death within 30 days from COVID‐19 hospitalization was evaluated as well. The incidence (95% confidence interval [CI]) of the events analyzed was calculated as the total number of events per 10,000 subjects in each group.

## Results

**Fig 1:**
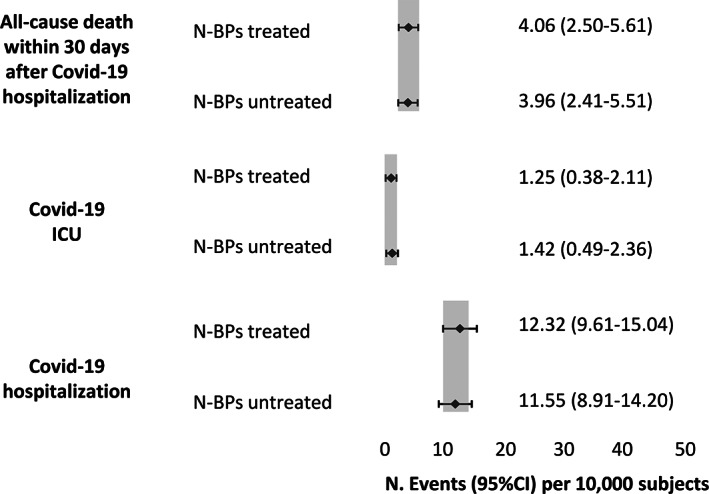
Incidence and 95% CI of COVID‐19–related events in N‐BPs treated and untreated subjects.

Within the sample population, around 64,000 subjects with bisphosphonates prescription, all N‐BPs, were identified. Most individuals were on alendronate (45.0%) or alendronate in combination with cholecalciferol (25.2%), 23.1% were on risedronate and 6.8% were on oral ibandronate. After matching by clinical and demographic characteristics, 63,185 subjects were included in each N‐BPs treated and untreated cohort (Table 1). A mean age of 73 years was observed. Patients were predominantly women (7.1% of male). Around 28% of individuals received corticosteroids, and 1.1% other anti‐osteoporotic treatments. N‐BP users were treated for a mean ± SD time of 4.4 ± 3.1 years; specifically, 60.3% of them received N‐BP for >3 years, 21.8% for 1–3 years, and 17.9% for <1 year. The same proportions of treatment duration were observed among N‐BP patients with COVID‐19 hospitalization (55.7% were prescribed N‐BP for >3 years, 31.7% for 1–3 years, and 12.6% <1 year). We found no difference in terms of incidence of COVID‐19 hospitalizations, COVID‐19 hospitalization in ICU, and mortality in oral N‐BPs users and nonusers. Figure 1 shows the incidence (*n*/10.000 subjects and 95% CI) of COVID‐19–related events during the first wave: specifically, incidence of COVID‐19 hospitalization was 12.32 (95% CI, 9.61–15.04) and 11.55 (95% CI, 8.91–14.20), of COVID‐19 hospitalization in ICU was 1.25 (95% CI, 0.38–2.11) and 1.42 (95% CI, 0.49–2.36), and of all‐cause death was 4.06 (95% CI, 2.50–5.61) and 3.96 (95% CI, 2.41–5.51) for oral N‐BPs users and nonusers, respectively.

**Table 1:** Descriptive Characteristics of the Overall 1:1 Matched Cohort

Characteristic	N‐BPs untreated cohort	N‐BPS treated cohort
*n*	63,185	63,185
Age (years), mean ± SD	73.4 ± 10.7	73.4 ± 10.7
Male, *n* (%)	4507 (7.1)	4507 (7.1)
Respiratory drugs, *n* (%)	14321 (22.7)	14321 (22.7)
Respiratory hospitalization, *n* (%)	5628 (8.9)	5628 (8.9)
Neoplasms, *n* (%)	4918 (7.8)	4918 (7.8)
CV hospitalization, *n* (%)	11704 (18.5)	11704 (18.5)
Antihypertensives, *n* (%)	41804 (66.2)	41804 (66.2)
Statins, *n* (%)	23830 (37.7)	23830 (37.7)
Antidiabetics, *n* (%)	7376 (11.7)	7376 (11.7)
Antidepressants, *n* (%)	11923 (18.9)	11923 (18.9)
Glucocorticoids, *n* (%)	17950 (28.4)	17950 (28.4)
Other anti‐osteoporotic drugs, *n* (%)	696 (1.1)	696 (1.1)

Antidiabetics included insulin treatment.

CV = cardiovascular; N‐BPs = amino‐bisphosphonates.

**Fig 2:**
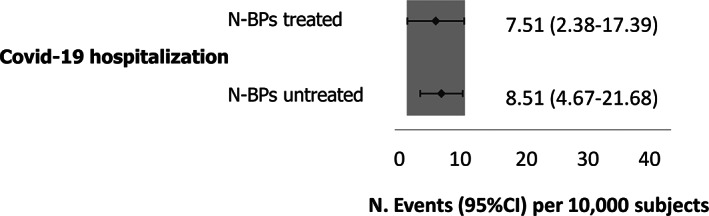
Incidence and 95% CI of COVID‐19 hospitalization in N‐BPs treated and untreated subjects with anti‐osteoporotic drugs and without corticosteroids.

**Fig 3:**
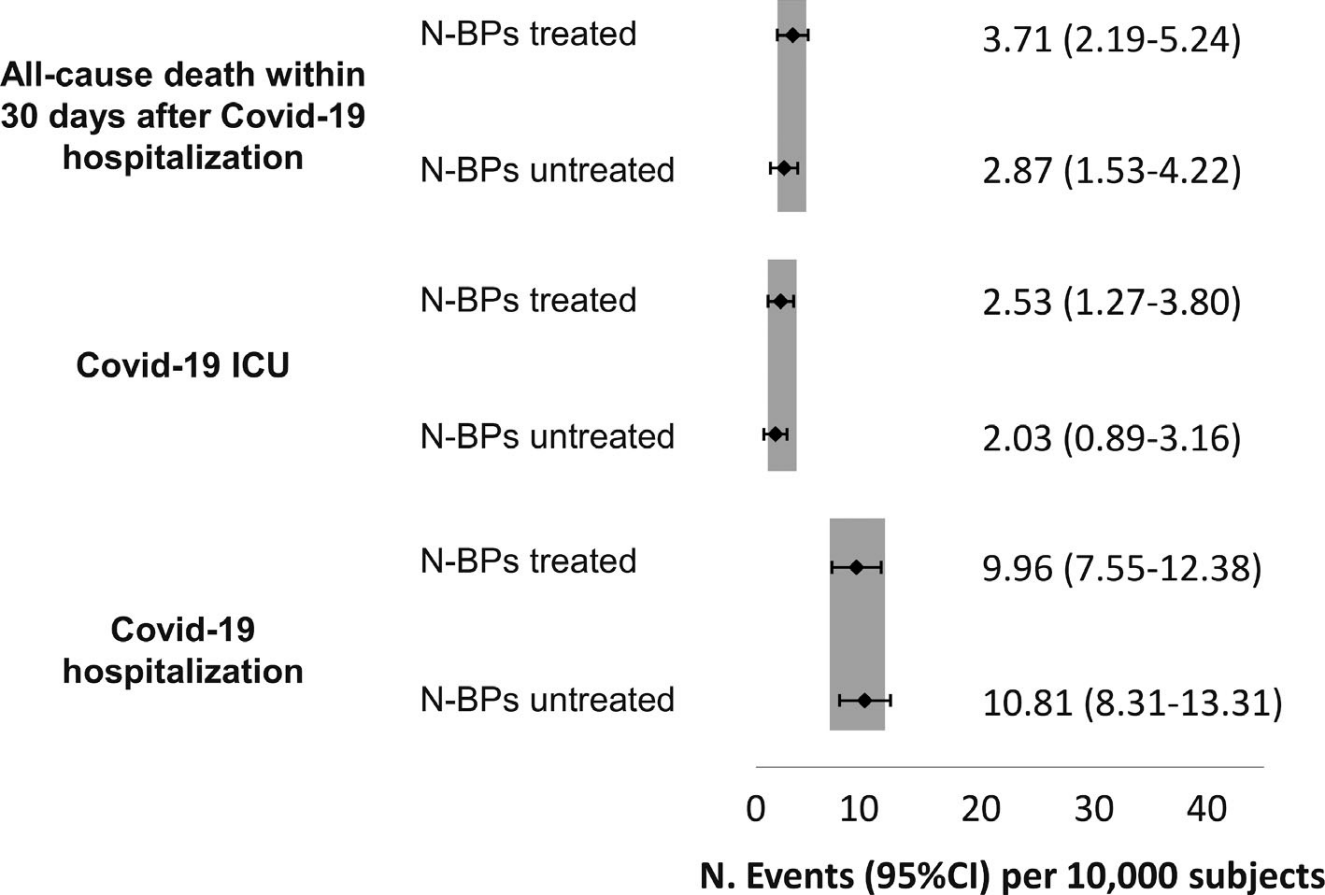
Incidence and 95% CI of COVID‐19–related events in N‐BPs treated and untreated without previous vertebral or hip fragility fractures.

**Fig 4:**
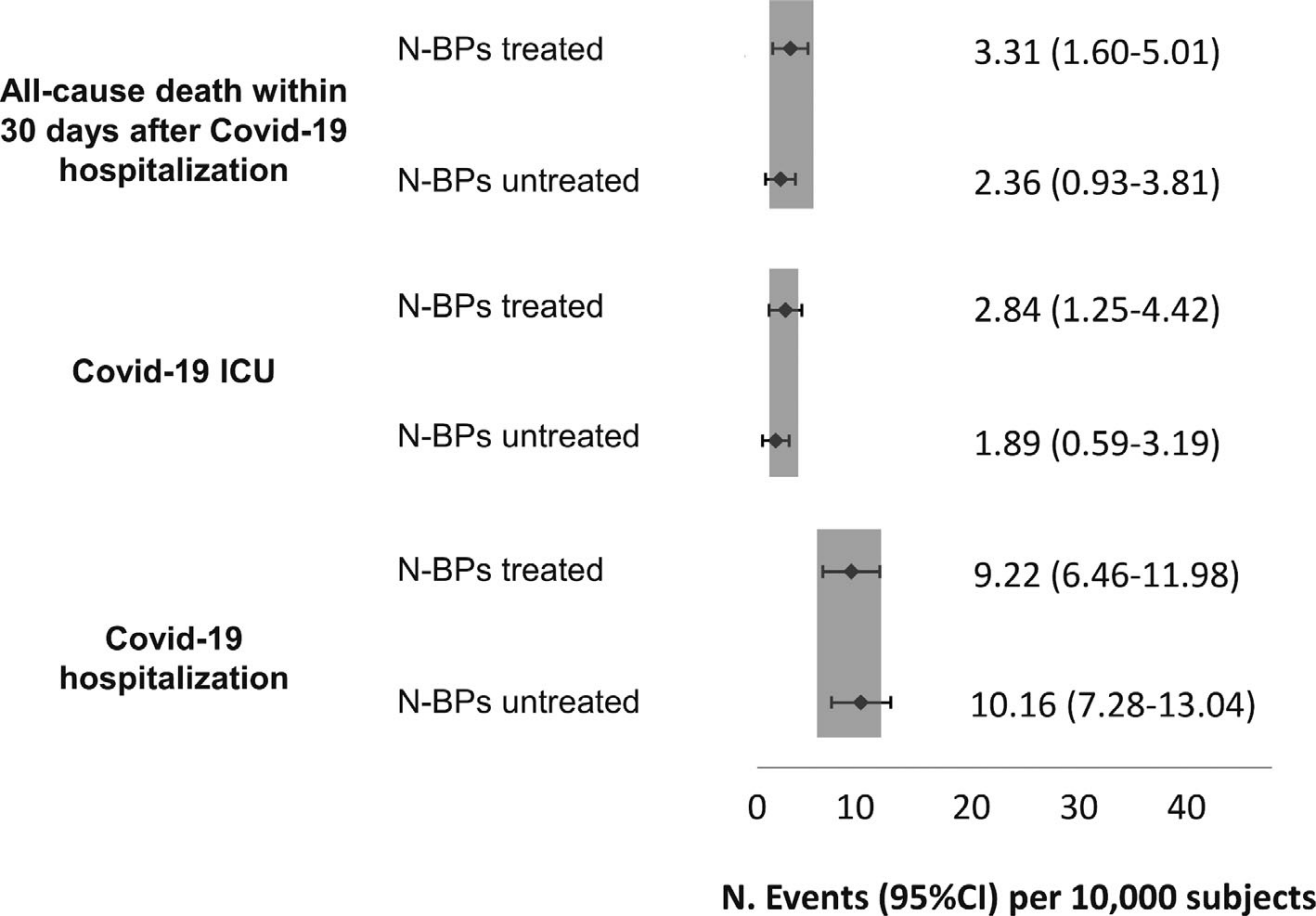
Incidence of COVID‐19–related events in bisphosphonates treated and untreated patients without previous vertebral or hip fracture without corticosteroids prescriptions.

Different scenarios were then investigated in further analyses. Patients with anti‐osteoporotic therapies, namely denosumab, teriparatide, calcitonin, and selective estrogen receptor modulators (SERMs), were matched 1:1 with N‐BPs users also receiving anti‐osteoporotic treatments, whereas corticosteroid users were not included. A total of 6065 subjects were included in each group (mean age 73.8 years, 4.5% male); their characteristics are reported in Table 2. We found no difference in COVID‐19 hospitalization incidence in N‐BPs users compared with other anti‐osteoporotic drug users (Fig. 2). Because of the low number of patients with events, incidence of COVID‐19 hospitalization in ICU and death was not analyzed. Sensitivity analyses conducted on patients without prevalent vertebral or hip fragility fractures (Fig. 3) and without concomitant glucocorticoid treatment (Fig. 4) yielded similar results.

**Table 2:** Characteristics of Patients With Other Antiosteoporotic Therapies and Without Corticosteroids Treated and Untreated With N‐BPs (1:1 Matched Cohorts)

	Patients with other antiosteoporotic therapies and without corticosteroids	
Characteristic	Other antiosteoporosis	Bisphosphonates
*n*	6065	6065
Age (years), mean ± SD	73.8 ± 10.7	73.8 ± 10.7
Male, *n* (%)	275 (4.5)	275 (4.5)
Respiratory drugs, *n* (%)	1019 (16.8)	1019 (16.8)
Respiratory hospitalization, *n* (%)	411 (6.8)	411 (6.8)
Neoplasms, *n* (%)	1000 (16.5)	1000 (16.5)
CV hospitalization, *n* (%)	1136 (18.7)	1136 (18.7)
Antihypertensives, *n* (%)	3827 (63.1)	3827 (63.1)
Statins, *n* (%)	2033 (33.5)	2033 (33.5)
Antidiabetics, *n* (%)	508 (8.4)	508 (8.4)
Antidepressants, *n* (%)	1152 (19.0)	1152 (19.0)

CV = cardiovascular.

## Discussion

We conducted a retrospective analysis on a nationwide cohort aimed to describe the incidence of COVID‐19 in patients treated with oral N‐BPs. We analyzed a representative sample of the Italian population treated with oral N‐BPs and compared with a 1:1 matched population of subjects not on N‐BPs. We found that the incidence of COVID‐19 hospitalization, ICU utilization, and COVID‐19 potentially related mortality were similar across groups and no differences were observed versus other anti‐osteoporotic drugs.

The cellular pattern underpinning the development of severe complications of COVID‐19 is still debated and controversial, but there is growing evidence that host response plays a fundamental role.^(^ ^7^ ^)^ In particular, immune cells that resides in the lungs, such as alveolar macrophages, might overreact to SARS‐CoV‐2 infection and lead to ARDS.^(^ ^8^ ^)^ Uncovering the factors associated with poor or good outcomes of SARS‐CoV‐2 infection is important in order to target interventions to those at higher risk of experiencing severe COVID‐19. Sing and colleagues^(^ ^9^ ^)^ demonstrated that N‐BPs were associated with a lower risk of pneumonia mortality, and there are hints that their administration might be protective even for severe SARS‐CoV‐2 infection.^(^ ^6^ ^)^ Preclinical and clinical studies had shown that bisphosphonates are capable to reduce the number of macrophages and γδ T‐cells which might reduce inflammation in targeted tissues, such as the lungs.^(^ ^10^, ^11^, ^12^ ^)^ Indeed, γδ T‐cells, migrated into the lungs, can recruit and activate neutrophils in response to a viral infection, such as SARS‐CoV‐2 and influenza.^(^ ^7^, ^13^, ^14^, ^15^ ^)^ γδ T‐cells' peripheral migration increases with aging and this phenomenon is probably at the basis of the otherwise known inflammaging.^(^ ^16^ ^)^ However, N‐BPs can acutely activate γδ‐T cells and promote their homing in on target tissue; this phenomenon is at the basis of their possible anti‐tumoral effect.^(^ ^17^, ^18^, ^19^ ^)^ However, prior exposure of N‐BPs was shown to be the most important clinical factor predicting the capacity of generating large numbers of γδ‐T cells.^(^ ^19^ ^)^ Moreover, the continuous presence of N‐BPs is toxic for T cells and blocks their proliferation.^(^ ^17^ ^)^ In summary, N‐BPs can expand and activate γδ‐T cell populations acutely (an effect that nicely explains the acute phase reaction seen after their administration); in contrast, chronic administration of N‐BPs can reduce the amount of circulating γδ‐T cell populations, with possible beneficial anti‐inflammatory effects. Another potential mechanism that might link bisphosphonates use with lower incidence of severe pulmonary manifestations of COVID‐19 is the demonstrated in vitro ability of N‐BPs of killing influenza‐infected cells, inhibiting viral replication and reducing the severity of influenza mortality.^(^ ^20^ ^)^ Furthermore, bisphosphonates inhibit the mevalonate pathway,^(^ ^21^ ^)^ which has been implicated in the development of hyperinflammation through the activation of neutrophils.^(^ ^22^ ^)^ Interestingly, N‐BPs have been postulated to inhibit the prenylation of G protein guanosine triphosphatases (Rab GTPases) with a possible anti‐viral effect, which might, eventually, prevent viral replication and spread.^(^ ^23^ ^)^ All this evidence provides a solid rationale for our study.

However, even if the biological rationale was plausible and the previous evidence indicated a possible protective role of bisphosphonates in preventing SARS‐CoV‐2 infection and/or severe COVID‐19 manifestations, we did not find any significant association in our nationwide cohort of patients exposed to oral N‐BPs. First, and of the utmost importance, our study is in line with previous findings from a small observational study that found oral N‐BPs to be safe in terms of SARS‐CoV‐2 infection risk,^(^ ^6^ ^)^ and that outcomes and unnecessary discontinuations should be avoided. We confirmed such a finding using a large real‐life data base. Furthermore, we confirmed our results also in patients treated with N‐BPs for primary prevention (ie, without prevalent vertebral or hip fractures) and patients not on chronic glucocorticoids. In addition, the incidence of COVID‐19 was similar in patients treated with N‐BPs as compared to other anti‐osteoporotic medications.

There are several possible explanations of our findings. First, patients on zoledronic acid, whose administration is limited to hospitalization in Italy, were not considered in the analysis due to limited data availability. Such patients, given the higher relative potency and immunological effects of zoledronic acid, might have been the ones with the largest protective effect on severe COVID‐19; indeed, zoledronic acid users might have experienced a differential response with regard to COVID‐19 infection and outcomes. Second, given the nature of the data source we did not have access to many covariates that may have influenced the primary outcome, such as unrecognized radiological vertebral fractures with relevant kyphosis, or the compliance of patients to the medications. However, in order to attenuate confounders, we designed a matching analysis that controlled for many clinically relevant covariates.

A key strength of our study is the generalizability of the results. Indeed, we have selected a large cohort of patients from the Italian population of bisphosphonate users and compared with a 1:1 matched cohort of nonusers. However, we should highlight some limitations common to all administrative claims data. For example, we had access to data regarding comorbidities, but we cannot extract the exact treatment applied. Nevertheless, cohort matching should have attenuated the possible confounding. Another example, as mentioned before, prescription claims do not indicate the actual use of the medication, possibly introducing a confounding by indication bias. Indeed, such bias could have induced spurious associations between N‐BPs administration and COVID‐19 outcomes, with an uncertain direction of effect. Moreover, we did not have access to clinical and laboratoristic data of COVID‐19 patients, hence we cannot rule out a possible effect of bisphosphonates on laboratoristic hyperinflammation (ie, C‐reactive protein serum levels on admission). Moreover, we observed a disparity in treatment length among patients treated with N‐BPs. Nevertheless, we did have access to ICU utilization during hospitalization and mortality related to COVID‐19, which are good proxies for the severity of the disease.

In summary, we analyzed the incidence of COVID‐19 among bisphosphonate users. We found that oral N‐BPs, mainly alendronate, were not statistically significantly associated with lower risk of severe COVID‐19; however, they did not seem to increase the risk. Further studies on intravenous N‐BPs, such as zoledronic acid, are needed to better ascertain the role of such potent medication on the risk of severe COVID‐19.

## Disclosures

All authors have completed the ICMJE uniform disclosure (available on request from the corresponding author) and declare no conflicts of interest.

## Compliance with ethical standard

All patients were identified in databases using an anonymous code and data were extracted and anonymized according to the Legislative Decree 196/03 and to the European General Data Protection Regulation (GDPR) (2016/679). Results derived from all analyses were produced as aggregated summaries which were not possible to link directly or indirectly to individual patients. Informed consent was not required because obtaining informed consent is impossible for organizational reasons (pronouncement of the Data Privacy Guarantor Authority, General Authorisation for personal data treatment for scientific research purposes – n.9/2014). Local ethics committee of all LHUs involved in the study was notified, according to Italian law.

## Patient and public involvement statement

This research was done without patient involvement. Patients were not invited to comment on the study design and were not consulted to develop patient relevant outcomes or interpret the results. Patients were not invited to contribute to the writing or editing of this document for readability or accuracy.

## Transparency declaration

The lead author (GA) (the manuscript's guarantor) affirms that the manuscript is an honest, accurate, and transparent account of the study being reported; that no important aspects of the study have been omitted; and that any discrepancies from the study as planned (and, if relevant, registered) have been explained.

## Author Contributions

Luca Degli Esposti: data curation; formal analysis; investigation; methodology; writing original draft; writing review editing. Valentina Perrone: formal analysis; methodology; writing review editing. Diego Sangiorgi: formal analysis; methodology; writing review editing. Margherita Andretta: data curation; writing review editing. Fausto Bertolini: data curation; writing review editing. Arturo Cavaliere: data curation; writing review editing. Andrea Ciaccia: data curation; writing review editing. Stefania Dell'orco: data curation; writing review editing. Stefano Grego: data curation; writing review editing. Loredana Ubertazzo: data curation; writing review editing. Adriano Vercellone: data curation; writing review editing. Davide Gatti: writing review editing. Angelo Fassio: writing review editing. Ombretta Viapiana: writing review editing. Maurizio Rossini: conceptualization; supervision; validation; writing review editing. Giovanni Adami: conceptualization; investigation; writing original draft; methodology; writing review editing.

### Peer Review

The peer review history for this article is available at https://publons.com/publon/10.1002/jbmr.4419.

## Data Availability

The data that support the findings of this study are available from the corresponding author upon reasonable request.
